# Identifying Motor Control Strategies and Their Role in Low Back Pain: A Cross-Disciplinary Approach Bridging Neurosciences With Movement Biomechanics

**DOI:** 10.3389/fpain.2021.715219

**Published:** 2021-08-11

**Authors:** Stefan Schmid, Christian Bangerter, Petra Schweinhardt, Michael L. Meier

**Affiliations:** ^1^Spinal Movement Biomechanics Group, Division of Physiotherapy, Department of Health Professions, Bern University of Applied Sciences, Bern, Switzerland; ^2^Faculty of Medicine, University of Basel, Basel, Switzerland; ^3^Department of Chiropractic Medicine, Balgrist University Hospital, University of Zurich, Integrative Spinal Research, Zurich, Switzerland; ^4^University of Zurich, Zurich, Switzerland; ^5^Alan Edwards Center for Research on Pain, McGill University, Montreal, QC, Canada

**Keywords:** low back pain, kinematics, pain-related fear, motor control, functional magnetic resonance imaging

## Abstract

Persistent low back pain (LBP) is a major health issue, and its treatment remains challenging due to a lack of pathophysiological understanding. A better understanding of LBP pathophysiology has been recognized as a research priority, however research on contributing mechanisms to LBP is often limited by siloed research within different disciplines. Novel cross-disciplinary approaches are necessary to fill important knowledge gaps in LBP research. This becomes particularly apparent when considering new theories about a potential role of changes in movement behavior (motor control) in the development and persistence of LBP. First evidence points toward the existence of different motor control strategy phenotypes, which are suggested to have pain-provoking effects in some individuals driven by interactions between neuroplastic, psychological and biomechanical factors. Yet, these phenotypes and their role in LBP need further validation, which can be systematically tested using an appropriate cross-disciplinary approach. Therefore, we propose a novel approach, connecting methods from neuroscience and biomechanics research including state-of-the-art optical motion capture, musculoskeletal modeling, functional magnetic resonance imaging and assessments of psychological factors. Ultimately, this cross-disciplinary approach might lead to the identification of different motor control strategy phenotypes with the potential to translate into clinical research for better treatment options.

## Introduction

Low back pain (LBP) is one of the most common conditions regarding years living with a disability throughout the world (1). The prevailing form of LBP does not have a clearly identifiable nociceptive source and is termed non-specific LBP (2). While many of these cases resolve within the first year, some still experience pain 1 year after onset, i.e., they develop a recurrent or chronic form, resulting in an enormous individual, economic and societal burden (1, 3). The clinical management of LBP is often limited to symptomatic interventions addressing the pain and its consequences, whereby effect sizes for these interventions are only low to moderate (2, 4, 5). This spurs a call for re-examining and identifying novel mechanisms associated with the development and persistence of LBP.

So far, research on LBP has identified several pathogenic mechanisms involving biophysical, genetic, social and psychological contributors (6). Research on LBP-related factors has revealed both biological and behavioral changes. On a biological level, LBP has been linked to disc degeneration, inflammation, and atrophy, fat infiltration and fiber type transition of paraspinal muscles (7–9). On a behavioral level, LBP has been shown to be associated with changes in movement, which can be described as changes in motor control (thereby affecting spine posture, stability, and movement) observed at the level of the nervous system [spinal- (10) and supraspinal (11) processes] as well as the musculoskeletal system (biomechanical mechanisms including muscle activity and kinematics) (12). Furthermore, psychological factors constitute important and non-negligible risk factors for the development and persistence of LBP (13).

However, as recently stated, research on these different pathomechanisms of LBP is often limited by significant knowledge gaps arising from siloed research within different research disciplines, highlighting the need for cross-disciplinary approaches that have the potential to identify important interactions between different mechanisms contributing to LBP (14). This becomes particularly evident when considering new theories about the role of subject-specific motor control strategies in LBP (movement behavior phenotypes which can predispose to and result from pain/injury) with potential long term consequences (12, 15, 16). In this context, LBP-associated changes in motor control are suggested to exert polydirectional and pain-provoking effects, involving interactions between neuroplastic, psychological and biomechanical factors that have not yet been systematically validated (15–17). Hence, to study such interactions and their role in the development and persistence of LBP, an appropriate cross-disciplinary approach that incorporates methods from neuroscience and movement biomechanics research is required.

Therefore, after a summary of the relevant literature, we present a novel cross-disciplinary approach combining neuroscientific and movement biomechanics research methods with the aim of identifying different motor control strategy phenotypes and their role in LBP as well as their underlying supraspinal, psychological, and biomechanical features. Ultimately, this approach might help to fill important knowledge gaps in LBP research with the potential to translate into clinical research for better treatment options.

## Biomechanical Mechanisms

Numerous studies have investigated biomechanical alterations in LBP, mainly by observing spine/trunk kinematics and muscle activity during functional activities as well as during steadily held postures with and without experimentally induced perturbations. Investigations of functional activities in LBP patients compared to healthy controls indicate trends toward a reduced lordotic posture and range of motion (RoM) in the lumbar spine during activities such as standing, walking, running, chair rising or picking up an object (18–20). In terms of muscle activity, studies show less clear trends, but instead a large variety of muscle activity patterns, ranging from higher lumbar extensor muscle activity to no differences or even lower activity in LBP patients compared to healthy controls (21). Studies combining kinematic and electromyographic experiments with musculoskeletal modeling report higher lumbar spine loading in LBP patients, which can be mainly explained by postural adaptations and increased trunk muscle activity (22, 23). Postural control studies with LBP patients revealed a delay in trunk muscle activity onset in response to both predictable and unpredictable perturbations (24, 25). These findings indicate that LBP patients experience a variety of motor control impairments, likely due to interaction deficiencies between sensory and motor systems that are responsible for goal-oriented spine posture, stability and movement (26, 27). Due to the large inter-individual variation, especially in terms of muscle activity patterns, van Dieën et al. (12) suggested that this might reflect the existence of multiple motor control strategies along a spectrum between two distinct phenotypes, resulting from adaptations in motor control to LBP and interference of LBP with motor control. Although not systematically tested yet, the “tight control” phenotype is suggested to involve increased trunk muscle excitability to provide tight control over trunk movements at the cost of higher tissue loading, whereas the “loose control” phenotype is characterized by a reduced excitability of trunk muscles to avoid high tissue loading at the cost of loose control over movement (12). Both motor control phenotypes might also be associated with supraspinal adaptions (e.g., cortical reorganization) (16), due to e.g., less dynamic motor behavior and impaired sensory feedback.

## Supraspinal Processes

More than 20 years ago and using magnetencephalography, researchers detected a shifted sensory representation of tactile input from the back in chronic LBP patients in the primary somatosensory cortex (28). Moreover, changes of paraspinal muscle representations in the primary motor cortex have been observed in chronic LBP patients, i.e., the motor cortex representations of the longissimus and deep multifidus muscles showed increased overlap compared to healthy controls, suggesting less fine-grained (“smudging”) cortical representations of paraspinal muscles (29). Such changes in the cortical organization of paraspinal muscles have also been shown to be associated with delayed activation of the transversus abdominis during rapid arm movements in patients with recurrent LBP, indicating a relationship between brain changes and motor control in LBP (11). However, it is still unclear whether the observed cortical sensorimotor changes in chronic LBP represent an epiphenomenon, simply triggered by altered sensory input [in particular from muscle spindles, the main transmitters of proprioceptive information (30)] and altered motor output, or if they are causally involved in the occurrence of recurrent and chronic LBP. The primary somatosensory cortex is well-known for encoding sensory aspects of pain (31) and recent research indicates that this region is hyperactive in chronic pain conditions, potentially driven by long-lasting disinhibition as shown in animal models of chronic pain and in humans (32, 33) Hence, the alterations in the primary somatosensory cortex in chronic LBP patients could be causally related to the experience of persistent LBP. Alternatively, the observed cortical sensorimotor changes might indirectly provoke persistent LBP by a reduced ability to (top-down) control paraspinal muscles. This might limit trunk movement variability and therefore spinal load distribution with unfavorable biomechanical and pro-nociceptive consequences such as increased loading on spinal tissues (12, 15). Indeed, current evidence suggests an association between brain changes and altered motor control in chronic LBP (34), which should be further explored to disentangle potential clinically relevant interactions between brain mechanisms and dysfunctional motor control strategies in LBP. Yet, while extensive knowledge exits about the cortical representation of various body parts and their potential reorganization based on environmental changes [e.g., the somatotopic representation of the hand and digits (35) and their cortical arrangement based on everyday hand use (36)], very little is known about a potential cortical topographic organization of sensory afferents from the back (e.g., along the thoracolumbar axis). In 2018, intra-cortical stimulation of the primary somatosensory cortex revealed the sensory representations of the thorax and abdomen (37) but still, the cortical representation of the back along the thoracolumbar axis, and in particular of proprioceptive afferents, is unclear. With regards to this, reorganization of proprioceptive input from paraspinal muscles is likely to be more important pathophysiologically for the chronification of LBP [compared to tactile input (38)], but the cortical somatotopy of proprioceptive input from the back has not yet been studied. Detailed cortical maps of paraspinal afferent input might therefore be of major importance to further explore potential relationships between brain changes and unfavorable motor control strategies (e.g., tight control strategy) in LBP.

## Psychological Factors

Pain-related fear and associated avoidance behavior as well as depression and anxiety have received extraordinary attention in the last two decades because they were empirically identified as important psychological factors in the development and persistence of LBP (3, 8, 39, 40). According to the Fear Avoidance model (41), misinterpretations of pain as a sign of harm in combination with negative affectivity and pain catastrophizing can lead to pain-related fear and avoidance behavior which might further aggravate pain, disability and depression (8). Indeed, positive relationships between pain-related fear, LBP intensity and disability have been found in systematic reviews and meta-analyses (39, 42), and fear avoidance beliefs have been shown to be associated with poor treatment outcome in patients suffering from LBP within a time period of <6 months (43). However, the predictive value of pain-related fear regarding the development of LBP is limited (39) and psychological factors in general (when considered in isolation) explain only a small proportion in outcomes such as pain intensity (44, 45). Yet, recent research has shown an association between pain-related fear and dysfunctional motor behavior in LBP patients and healthy individuals (46–48), indicating significant interactions between psychological factors and motor control (psychomotor interactions), which can promote potential clinically relevant consequences such as limited motor variability, increased paraspinal muscle co-contraction and loading on spinal tissues (15). Research on the role of pain-related fear in LBP should therefore systematically involve measures of motor control (such as spinal movement biomechanics) to identify potential pain-provoking interactions. With regards to this, a recently published meta-analysis including 52 studies found that higher levels of pain-related fear, catastrophizing and depression were significantly associated with reduced amplitudes of spinal movement and larger muscle activity, independently from pain intensity (49). Due to rather small effect sizes, however, it was concluded that more experimental studies with more specific and individualized measures of psychological factors, pain intensity, and spinal motor behavior are needed to better understand the underlying psychomotor interactions and to inform current treatment strategies.

## Building Bridges: A Cross-Disciplinary Approach

**Figure 1:**
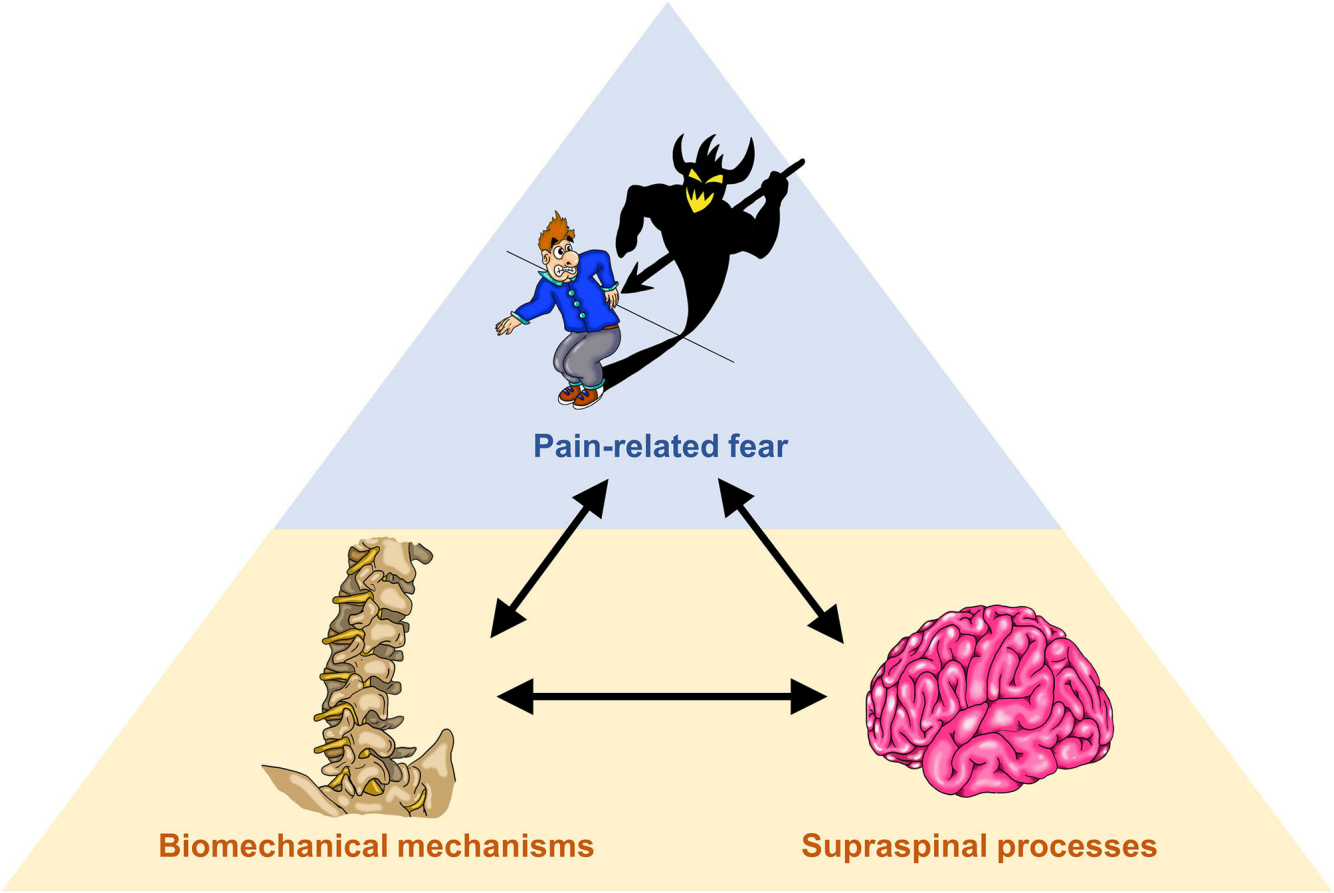
Illustration of interactions between pain-related fear (upper blue shaded area), biomechanical mechanisms and supraspinal processes (motor control; lower orange shaded area).

To investigate potential interactions between psychological factors, biomechanical mechanisms and supraspinal processes in LBP (Figure 1), we propose a cross-disciplinary approach, aiming at bridging between the “silos” neurosciences and movement biomechanics. The methodological basis comprises the assessment of psychological factors through questionnaires, biomechanical assessments of movement during functional activities based on high-resolution optical motion capturing and musculoskeletal modeling as well as the establishment of cortical topographic maps of paraspinal afferent input using functional magnetic resonance imaging (fMRI).

### Questionnaires

To assess pain-related fear, self-reports are an adequate direct measure of subjective feelings of fear that are easily accessible for clinicians and researchers (50). The most common self-reporting tools for assessing pain-related fear are questionnaires based on psychological constructs such as fear of movement/(re)injury [Tampa Scale for Kinesiophobia, TSK (51)], perceived harmfulness of daily activities [Photograph Series of Daily Activities, PHODA (52)] or fear avoidance beliefs [Fear Avoidance Beliefs, FABQ (53)]. However, it must be noted that even though recent neuroscientific and biomechanical evidence supports the diversity of pain-related fear constructs (46, 48, 54), it is still unclear how specific the different questionnaires are in assessing the various psychological constructs (55). Combining these questionnaires with biomechanical and neuroscientific measures might lead to a better understanding of the underlying psychological constructs. In addition, to reveal potential commonalities or differences between pain-related fear and general anxiety, the State-Trait Anxiety Inventory questionnaire (STAI) will be used to assess the participants' current level of anxiety (S-Anxiety) as well as aspects of “anxiety proneness” in general (T-Anxiety) (56, 57). To assess depressive symptoms, the Patient Health Questionnaire (PHQ-9) will be used (58).

### Assessing the Biomechanics of Spinal Movement

**Figure 2:**
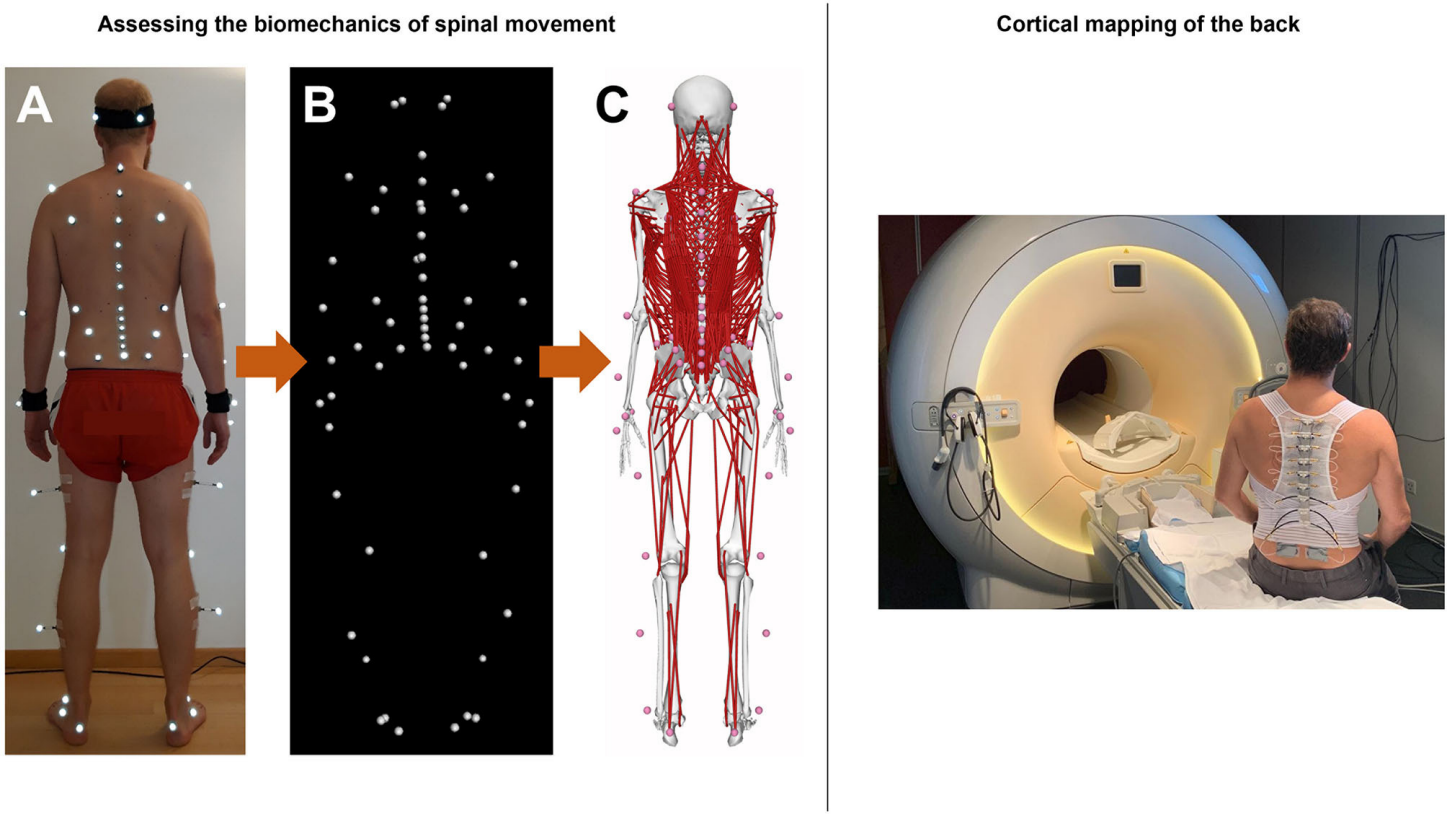
Left: Experimental and computational approach for quantifying movement biomechanics during functional activities. **(A)** Application of retro-reflective skin markers in a full-body configuration. **(B)** Capturing marker trajectories using infrared camera-based motion capture system. **(C)** Motion data-driven musculoskeletal full-body model with a detailed thoracolumbar spine. Right: Illustration of a subject wearing pneuVID elements in a MR environment. PneuVID can apply computer-controlled vibrotactile stimuli between 10 and 150Hz (and amplitudes 0.5–1mm) to a customizable stimulation area between 1 and 4 cm^2^ of each vibration unit. The vibration device controller (not shown) allows bilateral or unilateral vibrotactile stimulations of different body parts, including paraspinal tissue, in various stimulation settings.

The functional biomechanics of the spine are investigated using a comprehensive non-invasive experimental and computational approach, which combines state-of-the-art optical motion capture with advanced musculoskeletal modeling. Motion data are collected in a motion analysis laboratory, where participants are equipped with 58 retro-reflective skin markers according to a previously developed configuration (59) (Figure 2) and asked to perform various activities of daily living. These include walking and running on a level ground, climbing up and down a 5-step staircase, standing up from and sitting down on a chair, lifting up and putting down a 5 kg-box as well as performing vertical jump maneuvers. A 27-camera Vicon motion capture system and several force plates are used to record three-dimensional marker trajectories and ground reaction forces (GRFs), respectively (Figure 2). The suitability of this method for quantifying spinal motion during functional activities, which was previously used to investigate three-dimensional spinal kinematics in healthy populations as well as various patient populations including non-specific chronic LBP (20) was supported by comprehensive investigations of validity as well as within- and between-session reliability (60, 61).

For estimating intersegmental kinematics and spinal loading, we developed male and female musculoskeletal full-body models with a highly detailed spine (Figure 2) using the OpenSim modeling environment (62). To account for individual subject characteristics, the models are adjusted for each participant by considering segmental lengths and masses as well as sagittal plane spinal shape derived from the skin markers. Simulations are driven by the marker trajectories and GRFs collected in the motion analysis laboratory. Initial predictions of spinal loading in healthy pain-free individuals showed high consistency with reported *in vivo* measurements (62), supporting the suitability of this approach for accurately investigating LBP-related biomechanical adaptations in large patient populations.

To account for LBP-related changes in muscle activity, we are planning to include electromyographic (EMG) measurements of the main trunk stabilizers and to use this information as additional input for our models. This will further increase prediction accuracy, especially when participants present activity patterns such as increased antagonistic muscle coactivation, which was shown to have direct implications on spinal loading (22, 23).

### Cortical Mapping of the Back

Non-invasive human brain imaging techniques such fMRI with its high spatial resolution provide suitable tools for the investigation of the cortical representation of different body parts (63). We developed a novel MR-compatible vibration device (pneumatic spinal vibration device, pneuVID, Figure 2), which can apply computer-controlled vibrotactile stimuli between 10 and 150 Hz to different thoracolumbar segmental levels. This is the first apparatus specifically designed for paraspinal muscle vibration on different segmental levels in an MR environment. The pneuVID has been successfully tested for MR compatibility and permits MR measurements in supine position to allow better and more comfortable subject positioning (using special pillows for the back to embed the vibration units) and head fixation.

Using the pneuVID in combination with high spatial resolution fMRI (3 or 7 Tesla), detailed cortical maps of paraspinal afferent input can be explored using different vibration frequencies: Applying vibratory stimulation at frequencies between 60 and 80 Hz and amplitudes of 0.5–1 mm on paraspinal muscles has been shown to be a potent stimulus for muscle spindle activation (and therefore proprioceptive signaling) (26). In contrast, stimulus frequencies around 20 Hz will primarily activate receptors in superficial skin layers (e.g., Meissner's corpuscles) (64). Thus, by using randomized fMRI stimulation protocols including different vibration frequencies at various thoracolumbar segmental levels, the current approach has the potential to identify and differentiate cortical proprioceptive somatotopic maps from tactile somatotopic maps of the back and compare them between healthy controls and LBP patients of different symptom durations. It must be noted, however, that it is currently unclear which trunk muscle spindles are affected in their activation profiles by pneuVID stimulation. We assume that mainly superficial muscles along the thoracolumbar axis (i.e., longissimus and spinalis muscles) are targeted. Nonetheless, since the stimulation sites are also located over the rotatores and multifidi muscles, these structures, which are important in providing proprioceptive information [with the rotatores breves muscles having the highest density of muscle spindles of the lumbar and thoracic muscles (65)] might also be affected.

## Filling the Gaps

Using the methodologies spanning different research disciplines as described above, the current approach has the potential to address important questions in LBP research:

1. Do loose/tight motor control strategy phenotypes indeed exist and/or do other motor control strategies exist? Biomechanical assessments of dynamic movement tasks, involving subject-specific spine kinematics, segmental loadings and paraspinal muscle forces during daily activities (lifting, walking running etc.), will be performed to investigate potential relationships with LBP duration, disability, and psychological factors. Relevant features will be extracted for subsequent data analysis (e.g., unsupervised cluster analysis) with the goal of classifying different motor control strategy phenotypes that are possibly associated with different LBP symptom durations (acute, subacute, and chronic stages).
2. Can a topographic cortical organization of thoracolumbar sensory input be identified? How does this cortical organization relate to the identified motor control strategy phenotypes in LBP? For example, it is plausible that degraded paraspinal proprioceptive feedback (e.g., provoked by a tight control strategy) is causally linked to LBP-provoking alterations in motor control *via* neuroplastic cortical changes (e.g., “smudging” of cortical maps of paraspinal afferent input) (16). For the first time, we therefore aim to test whether cortical maps of thoracolumbar afferent input demonstrate a relationship with spinal movement patterns, LBP duration and psychological factors. Novel insights into these relationships would pave the way for future investigations of causal interactions between cortical changes and motor control strategies using longitudinal study designs.

As recently stated, a better understanding of musculoskeletal pain depends on reconnecting the brain with the rest of the body (14). Our approach including investigations of potential interactions between supraspinal processes and biomechanical mechanisms contributes to this reconnection and could facilitate a transfer of the knowledge generated within the past 20 years of research on motor control related neuroplasticity into clinical practice.

## Clinical Impact

Provided that the suggested motor control strategy phenotypes can be reliably identified using the approach described in this article, the knowledge generated might lead to important implications for clinical research and interventions. For example, it has been proposed that a persistent “tight control strategy” may be specifically targeted by reducing muscle excitability and co-contraction while increasing movement variability in motor control exercise (12). With regards to this, our approach might provide promising behavior- and neuroimaging-based outcomes to test the potential therapeutic effect of individualized motor control exercises and how they compare to other treatment approaches.

## Data Availability Statement

The original contributions presented in the study are included in the article/supplementary material, further inquiries can be directed to the corresponding author.

## Author Contributions

SS and MM wrote the first draft of the manuscript. CB and PS critically revised the manuscript and contributed additional text parts. All the authors approved the version to be published.

## Conflict of Interest

The authors declare that the research was conducted in the absence of any commercial or financial relationships that could be construed as a potential conflict of interest.

## Publisher's Note

All claims expressed in this article are solely those of the authors and do not necessarily represent those of their affiliated organizations, or those of the publisher, the editors and the reviewers. Any product that may be evaluated in this article, or claim that may be made by its manufacturer, is not guaranteed or endorsed by the publisher.

## References

[1] Wu A March L Zheng X Huang J Wang X Zhao J. Global low back pain prevalence and years lived with disability from 1990 to 2017: estimates from the Global Burden of Disease Study 2017. Ann Transl Med. (2020) 8:299. 10.21037/atm.2020.02.175 32355743 PMC7186678

[2] Maher C Underwood M Buchbinder R. Non-specific low back pain. Lancet. (2017) 389:736–47. 10.1016/S0140-6736(16)30970-9 27745712

[3] Vlaeyen JWS Maher CG Wiech K van Zundert J Meloto CB Diatchenko L. Low back pain. Nat Rev Dis Primers Dis Primers. (2018) 4:1–18. 10.1038/s41572-018-0052-1 30546064

[4] Rubinstein SM Terwee CB Assendelft WJJ Boer MR de van Tulder MW. Spinal manipulative therapy for acute low back pain: an update of the cochrane review. Spine. (2013) 38:E158–77. 10.1097/BRS.0b013e31827dd89d 23169072

[5] van Middelkoop M Rubinstein SM Kuijpers T Verhagen AP Ostelo R Koes BW. A systematic review on the effectiveness of physical and rehabilitation interventions for chronic non-specific low back pain. Eur Spine J. (2011) 20:19–39. 10.1007/s00586-010-1518-3 20640863 PMC3036018

[6] Hartvigsen J Hancock MJ Kongsted A Louw Q Ferreira ML Genevay S. What low back pain is and why we need to pay attention. Lancet. (2018) 391:2356–67. 10.1016/S0140-6736(18)30480-X 29573870

[7] Goubert D van Oosterwijck J Meeus M Danneels L. Structural changes of lumbar muscles in non-specific low back pain: a systematic review. Pain Phys. (2016) 19:E985–1000. 10.36076/ppj/2016.19.E985 27676689

[8] Knezevic NN Candido KD Vlaeyen JWS van Zundert J Cohen SP. Low back pain. Lancet. (2021) 398:78–92. 10.1016/S0140-6736(21)00733-9 34115979

[9] Brinjikji W Diehn FE Jarvik JG Carr CM Kallmes DF Murad MH. MRI findings of disc degeneration are more prevalent in adults with low back pain than in asymptomatic controls: a systematic review and meta-analysis. AJNR Am J Neuroradiol. (2015) 36:2394–9. 10.3174/ajnr.A4498 26359154 PMC7964277

[10] Hodges PW Tucker K. Moving differently in pain: a new theory to explain the adaptation to pain. Pain. (2011) 152(3 Suppl):S90–8. 10.1016/j.pain.2010.10.020 21087823

[11] Tsao H Galea MP Hodges PW. Reorganization of the motor cortex is associated with postural control deficits in recurrent low back pain. Brain. (2008) 131(Pt 8):2161–71. 10.1093/brain/awn154 18669505

[12] van Dieën JH Reeves NP Kawchuk G van Dillen LR Hodges PW. Motor control changes in low back pain: divergence in presentations and mechanisms. J Orthop Sports Phys Ther. (2019) 49:370–9. 10.2519/jospt.2019.7917 29895230 PMC7393576

[13] Clays E Bacquer D de Leynen F Kornitzer M Kittel F Backer G de. The impact of psychosocial factors on low back pain: longitudinal results from the Belstress study. Spine. (2007) 32:262–8. 10.1097/01.brs.0000251884.94821.c0 17224824

[14] Langevin HM. Reconnecting the brain with the rest of the body in musculoskeletal pain research. J Pain. (2020) 22:1–8. 10.1016/j.jpain.2020.02.006 32553621 PMC7736274

[15] Meier ML Vrana A Schweinhardt P. Low back pain: the potential contribution of supraspinal motor control and proprioception. Neuroscientist. (2019) 25:583–96. 10.1177/1073858418809074 30387689 PMC6900582

[16] van Dieën JH Flor H Hodges PW. Low-back pain patients learn to adapt motor behavior with adverse secondary consequences. Exerc Sport Sci Rev. (2017) 45:223–9. 10.1249/JES.0000000000000121 28704216

[17] Hodges PW Smeets RJ. Interaction between pain, movement, and physical activity: short-term benefits, long-term consequences, and targets for treatment. Clin J Pain. (2015) 31:97–107. 10.1097/AJP.0000000000000098 24709625

[18] Christe G Redhead L Legrand T Jolles BM Favre J. Multi-segment analysis of spinal kinematics during sit-to-stand in patients with chronic low back pain. J Biomech. (2016) 49:2060–7. 10.1016/j.jbiomech.2016.05.015 27262182

[19] Gombatto SP D'Arpa N Landerholm S Mateo C O'Connor R Tokunaga J. Differences in kinematics of the lumbar spine and lower extremities between people with and without low back pain during the down phase of a pick up task, an observational study. Musculoskelet Sci Pract. (2017) 28:25–31. 10.1016/j.msksp.2016.12.017 28171775

[20] Simonet E Winteler B Frangi J Suter M Meier ML Eichelberger P. Walking and running with non-specific chronic low back pain: what about the lumbar lordosis angle? J Biomech. (2020) 108:109883. 10.1016/j.jbiomech.2020.109883 32635997

[21] van Dieën JH Selen LPJ Cholewicki J. Trunk muscle activation in low-back pain patients, an analysis of the literature. J Electromyogr Kinesiol. (2003) 13:333–51. 10.1016/S1050-6411(03)00041-5 12832164

[22] Marras WS Davis KG Ferguson SA Lucas BR Gupta P. Spine loading characteristics of patients with low back pain compared with asymptomatic individuals. Spine. (2001) 26:2566–74. 10.1097/00007632-200112010-00009 11725237

[23] Marras WS Ferguson SA Burr D Davis KG Gupta P. Spine loading in patients with low back pain during asymmetric lifting exertions. Spine J. (2004) 4:64–75. 10.1016/S1529-9430(03)00424-8 14749195

[24] MacDonald D Moseley GL Hodges PW. Why do some patients keep hurting their back? Evidence of ongoing back muscle dysfunction during remission from recurrent back pain. Pain. (2009) 142:183–8. 10.1016/j.pain.2008.12.002 19186001

[25] Prins MR Griffioen M Veeger TTJ Kiers H Meijer OG van der Wurff P. Evidence of splinting in low back pain? A systematic review of perturbation studies. Eur Spine J. (2018) 27:40–59. 10.1007/s00586-017-5287-0 28900711

[26] Claeys K Brumagne S Dankaerts W Kiers H Janssens L. Decreased variability in postural control strategies in young people with non-specific low back pain is associated with altered proprioceptive reweighting. Eur J Appl Physiol. (2011) 111:115–23. 10.1007/s00421-010-1637-x 20824281

[27] Hodges PW Cholewicki J van Dieën JH. Spinal Control: The Rehabilitation of Back Pain State of the Art and Science. Edinburgh: Elsevier (2013).

[28] Flor H Braun C Elbert T Birbaumer N. Extensive reorganization of primary somatosensory cortex in chronic back pain patients. Neurosci Lett. (1997) 224:5–8. 10.1016/S0304-3940(97)13441-3 9132689

[29] Tsao H Danneels LA Hodges PW. ISSLS prize winner: smudging the motor brain in young adults with recurrent low back pain. Spine. (2011) 36:1721–7. 10.1097/BRS.0b013e31821c4267 21508892

[30] Riemann BL Lephart SM. The sensorimotor system, part I: the physiologic basis of functional joint stability. J Athl Train. (2002) 37:71–9. 16558670 PMC164311

[31] Bushnell MC Duncan GH Hofbauer RK Ha B Chen JI Carrier B. Pain perception: is there a role for primary somatosensory cortex? Proc Natl Acad Sci U S A. (1999) 96:7705–9. 10.1073/pnas.96.14.7705 10393884 PMC33605

[32] Eto K Wake H Watanabe M Ishibashi H Noda M Yanagawa Y. Inter-regional contribution of enhanced activity of the primary somatosensory cortex to the anterior cingulate cortex accelerates chronic pain behavior. J Neurosci. (2011) 31:7631–6. 10.1523/JNEUROSCI.0946-11.2011 21613476 PMC6633125

[33] Lim M Roosink M Kim JS Kim DJ Kim HW Lee EB. Disinhibition of the primary somatosensory cortex in patients with fibromyalgia. Pain. (2015) 156:666–74. 10.1097/j.pain.0000000000000096 25630027

[34] Elgueta-Cancino E Schabrun S Hodges P. Is the organization of the primary motor cortex in low back pain related to pain, movement, and/or sensation? Clin J Pain. (2018) 34:207–16. 10.1097/AJP.0000000000000535 28719508

[35] Sutherling WW Levesque MF Baumgartner C. Cortical sensory representation of the human hand: size of finger regions and nonoverlapping digit somatotopy. Neurology. (1992) 42:1020–8. 10.1212/WNL.42.5.1020 1579225

[36] Ejaz N Hamada M Diedrichsen J. Hand use predicts the structure of representations in sensorimotor cortex. Nat Neurosci. (2015) 18:1034–40. 10.1038/nn.4038 26030847

[37] Roux F-E Djidjeli I Durand J-B. Functional architecture of the somatosensory homunculus detected by electrostimulation. J Physiol. (2018) 596:941–56. 10.1113/JP275243 29285773 PMC5830421

[38] Beaudette SM Larson KJ Larson DJ Brown SHM. Low back skin sensitivity has minimal impact on active lumbar spine proprioception and stability in healthy adults. Exp Brain Res. (2016) 234:2215–26. 10.1007/s00221-016-4625-5 27010722

[39] Martinez-Calderon J Flores-Cortes M Morales-Asencio JM Luque-Suarez A. Pain-related fear, pain intensity and function in individuals with chronic musculoskeletal pain: a systematic review and meta-analysis. J Pain. (2019) 20:1394–415. 10.1016/j.jpain.2019.04.009 31063874

[40] Ranger TA Cicuttini FM Jensen TS Manniche C Heritier S Urquhart DM. Catastrophization, fear of movement, anxiety, and depression are associated with persistent, severe low back pain and disability. Spine J. (2020) 20:857–65. 10.1016/j.spinee.2020.02.002 32045707

[41] Leeuw M Goossens MEJB Linton SJ Crombez G Boersma K Vlaeyen JWS. The fear-avoidance model of musculoskeletal pain: current state of scientific evidence. J Behav Med. (2007) 30:77–94. 10.1007/s10865-006-9085-0 17180640

[42] Zale EL Lange KL Fields SA Ditre JW. The relation between pain-related fear and disability: a meta-analysis. J Pain. (2013) 14:1019–30. 10.1016/j.jpain.2013.05.005 23850095 PMC3791167

[43] Wertli MM Rasmussen-Barr E Held U Weiser S Bachmann LM Brunner F. Fear-avoidance beliefs-a moderator of treatment efficacy in patients with low back pain: a systematic review. Spine J. (2014) 14:2658–78. 10.1016/j.spinee.2014.02.033 24614254

[44] Klyne DM van den Hoorn W Barbe MF Cholewicki J M Hall L Khan A. Cohort profile: why do people keep hurting their back? BMC Res Notes. (2020) 13:538. 10.1186/s13104-020-05356-z 33203448 PMC7672992

[45] Schweinhardt P. Where has the 'bio' in bio-psycho-social gone? Curr Opin Support Palliat Care. (2019) 13:94–8. 10.1097/SPC.0000000000000420 30893103

[46] Matheve T Baets L de Bogaerts K Timmermans A. Lumbar range of motion in chronic low back pain is predicted by task-specific, but not by general measures of pain-related fear. Eur J Pain. (2019) 23:1171–84. 10.1002/ejp.1384 30793429

[47] Geisser ME Haig AJ Wallbom AS Wiggert EA. Pain-related fear, lumbar flexion, and dynamic EMG among persons with chronic musculoskeletal low back pain. Clin J Pain. (2004) 20:61–9. 10.1097/00002508-200403000-00001 14770044

[48] Knechtle D Schmid S Suter M Riner F Moschini G Senteler M. Fear avoidance beliefs are associated with reduced lumbar spine flexion during object lifting in pain-free adults. Pain. (2020) 162:1621–31. 10.1101/2020.04.01.20049999 PMC8120682 33323888

[49] Christe G Crombez G Edd S Opsommer E Jolles BM Favre J. The relationship between psychological factors and spinal motor behaviour in low back pain: a systematic review and meta-analysis. Pain. (2020). 10.1097/j.pain.0000000000002065 33591109

[50] LeDoux JE Hofmann SG. The subjective experience of emotion: a fearful review. Curr Opin Behav Sci. (2018) 19:67–72. 10.1016/j.cobeha.2017.09.011 PMC11444666 39355512

[51] Houben RMA Leeuw M Vlaeyen JWS Goubert L Picavet HSJ. Fear of movement/injury in the general population: factor structure and psychometric properties of an adapted version of the Tampa Scale for Kinesiophobia. J Behav Med. (2005) 28:415–24. 10.1007/s10865-005-9011-x 16187010

[52] Leeuw M Goossens MEJB van Breukelen GJP Boersma K Vlaeyen JWS. Measuring perceived harmfulness of physical activities in patients with chronic low back pain: the Photograph Series of Daily Activities–short electronic version. J Pain. (2007) 8:840–9. 10.1016/j.jpain.2007.05.013 17632038

[53] Pfingsten M Kröner-Herwig B Leibing E Kronshage U Hildebrandt J. Validation of the German version of the Fear-Avoidance Beliefs Questionnaire (FABQ). Eur J Pain. (2000) 4:259–66. 10.1053/eujp.2000.0178 10985869

[54] Meier ML Vrana A Humphreys BK Seifritz E Stämpfli P Schweinhardt P. Pain-related fear-dissociable neural sources of different fear constructs. eNeuro. (2018) 5:ENEURO.0107–18.2018. 10.1101/251751 PMC6325558 30627654

[55] Lundberg M Grimby-Ekman A Verbunt J Simmonds MJ. Pain-related fear: a critical review of the related measures. Pain Res Treat. (2011) 2011:494196. 10.1155/2011/494196 22191022 PMC3236324

[56] Spielberger CD Gorsuch RL. Manual for the State-Trait Anxiety Inventory (Form Y): (“Self-Evaluation Questionnaire”). Palo Alto, CA: Consulting Psychologists Press, Inc. (1983).

[57] Julian LJ. Measures of anxiety: State-Trait Anxiety Inventory (STAI), Beck Anxiety Inventory (BAI), and Hospital Anxiety and Depression Scale-Anxiety (HADS-A). Arthritis Care Res. (2011) 63(Suppl 11):S467–72. 10.1002/acr.20561 PMC3879951 22588767

[58] Kroenke K Spitzer RL Williams JB. The PHQ-9: validity of a brief depression severity measure. J Gen Intern Med. (2001) 16:606–13. 10.1046/j.1525-1497.2001.016009606.x 11556941 PMC1495268

[59] Schmid S Bruhin B Ignasiak D Romkes J Taylor WR Ferguson SJ. Spinal kinematics during gait in healthy individuals across different age groups. Hum Mov Sci. (2017) 54:73–81. 10.1016/j.humov.2017.04.001 28410535

[60] Niggli LA Eichelberger P Bangerter C Baur H Schmid S. Between-Session Reliability of Skin Marker-Derived Spinal Kinematics During Functional Activities. (2020). Available online at: http://arxiv.org/pdf/2010.15956v1 10.1016/j.gaitpost.2021.02.008 33636456

[61] Zemp R List R Gülay T Elsig JP Naxera J Taylor WR. Soft tissue artefacts of the human back: comparison of the sagittal curvature of the spine measured using skin markers and an open upright MRI. PLoS ONE. (2014) 9:e0095426. 10.1371/journal.pone.0095426 PMC3991691 24748013

[62] Connolly LEP Schmid S Moschini G Meier ML Senteler M. Motion Capture-driven Musculoskeletal Spine Modeling: An OpenSim-based Inverse Kinematics Approach. (2021). Available online at: http://arxiv.org/pdf/2101.12272v1

[63] Nelson AJ Chen R. Digit somatotopy within cortical areas of the postcentral gyrus in humans. Cereb Cortex. (2008) 18:2341–51. 10.1093/cercor/bhm257 18245039

[64] Weerakkody NS Mahns DA Taylor JL Gandevia SC. Impairment of human proprioception by high-frequency cutaneous vibration. J Physiol. (2007) 581(Pt 3):971–80. 10.1113/jphysiol.2006.126854 17412774 PMC2170847

[65] Boucher J-A Abboud J Nougarou F Normand MC Descarreaux M. The effects of vibration and muscle fatigue on trunk sensorimotor control in low back pain patients. PLoS ONE. (2015) 10:e0135838. 10.1371/journal.pone.0135838 26308725 PMC4550235

